# Body composition and its association with fatigue in the first 2 years after colorectal cancer diagnosis

**DOI:** 10.1007/s11764-020-00953-0

**Published:** 2020-10-17

**Authors:** H. van Baar, M. J. L. Bours, S. Beijer, M. van Zutphen, F. J. B. van Duijnhoven, D. E. Kok, E. Wesselink, J. H. W. de Wilt, E. Kampman, R. M. Winkels

**Affiliations:** 1 Division of Human Nutrition and Health, Wageningen University & Research Wageningen, PO Box 17, 6700 AA Wageningen, The Netherlands; 2 Department of Epidemiology, GROW School for Oncology and Developmental Biology, Maastricht University, Maastricht, The Netherlands; 3 Department of Research & Development, Netherlands Comprehensive Cancer Organisation (IKNL), Utrecht, The Netherlands; 4 Department of Surgery, Radboud Universitair Medisch Centrum, Nijmegen, The Netherlands

**Keywords:** Fatigue, Colorectal cancer, Adipose tissue, Muscle mass, Fat mass, Skeletal muscle radiodensity

## Abstract

**Purpose:**

Persistent fatigue among colorectal cancer (CRC) patients might be associated with unfavorable body composition, but data are sparse and inconsistent. We studied how skeletal muscle index (SMI), skeletal muscle radiodensity (SMR), visceral adipose tissue (VAT), and subcutaneous adipose tissue (SAT) at diagnosis are associated with fatigue up to 24 months post-diagnosis in stage I–III CRC patients.

**Methods:**

SMI, SMR, VAT, and SAT were assessed among 646 CRC patients using pre-treatment computed tomography images. Fatigue at diagnosis, at 6, and 24 months post-diagnosis was assessed using the European Organization for Research and Treatment of Cancer Quality of Life Questionnaire. The association of SMI, SMR, VAT, and SAT with fatigue (yes/no) was assessed using confounder-adjusted restricted cubic spline analyses.

**Results:**

Prevalence of fatigue at diagnosis was 18%, at 6 months 25%, and at 24 months 12%. At diagnosis, a significant (*p* = 0.01) non-linear association of higher levels of SAT with higher prevalence of fatigue was observed. Lower levels of SMR were linearly associated with higher prevalence of fatigue at 6 months post-diagnosis (overall association *p* = 0.02). None of the body composition parameters were significantly associated with fatigue at 24 months.

**Conclusion:**

Having more SAT was associated with more fatigue at diagnosis, while low levels of SMR were associated with more fatigue at 6 months post-diagnosis.

**Implications for Cancer Survivors:**

Our results suggest that it may be interesting to investigate whether interventions that aim to increase SMR around the time of diagnosis may help to lower fatigue. However, more knowledge is needed to understand the mechanisms behind the association of SMR with fatigue.

**Electronic supplementary material:**

The online version of this article (10.1007/s11764-020-00953-0) contains supplementary material, which is available to authorized users.

## Introduction

Fatigue is highly prevalent among stage I–III colorectal cancer (CRC) patients. Fatigue is often already experienced before diagnosis [1] and can persist for years after treatment. The highest prevalence (up to 85%) of fatigue is seen during and shortly after treatment [2, 3], and although the prevalence decreases in the years after treatment, up to 40% of CRC patients experience fatigue in the first 5 years after diagnosis [4]. Fatigue is one of the most debilitating side effects of cancer and has a substantial negative impact on mood, work, social relationships, and overall quality of life [2].

Fatigue among cancer survivors is thought to have a multi-factorial etiology. Fatigue has been associated with treatment (radio- and chemotherapy), stage of disease, presence of (multiple) comorbidities, specific medications with sedating side effects, psychological factors (e.g., depression), decreased physical activity, and malnutrition [2, 5–7]. Several studies investigated whether there was an association between obesity and fatigue among cancer patients. Although some studies observed that obesity was associated with more fatigue [8–10], other studies did not find a significant association [11–13]. The use of body mass index (BMI) as measure of obesity might explain the mixed results in these studies: BMI does not take adipose tissue distribution into account and is an unreliable proxy for adiposity [14]. In cancer populations, computed tomography (CT) imaging is a key part of diagnosis and planning of treatment. Through those images, opportunistic imaging of skeletal muscle and adipose tissue became popular over the last two decades. These existing CT images are used to quantify visceral and subcutaneous adipose tissue (VAT and SAT respectively), skeletal muscle mass, and skeletal muscle radiodensity (SMR, indirect measurement of fat infiltration within the muscle cell, where lower levels of radiodensity indicate higher fat infiltration). Associations between CT-determined body composition and fatigue in cancer survivors have been studied in only a few studies, with mixed results. Two studies conducted among 734 stage IIIb/IV non-small cell lung cancer patients [15] and 151 advanced colorectal, breast, or prostate cancer patients [16], respectively, both observed that less skeletal mass at diagnosis was associated with higher levels of pre-treatment fatigue among men. Both studies did not observe significant associations among women. Another study conducted among 96 stage I–III CRC survivors who were on average 5.2 years post-diagnosis did not find an association between skeletal muscle mass at diagnosis and levels of fatigue at that point post-diagnosis [17]. The latter study also investigated VAT and SMR and did not find an association between those body composition parameters and fatigue [17]. The association between SMR at diagnosis and pretreatment fatigue was also investigated among the earlier quoted study among non-small cell lung cancer patients, and no significant association was observed there either [15]. To our knowledge, no study has investigated the association between SAT and fatigue among cancer patients.

The aim of the current study was to investigate the association of body composition parameters (Skeletal Muscle Index (SMI), SMR, VAT, and SAT) at diagnosis with fatigue at three timepoints (diagnosis, 6 months, and 24 months post-diagnosis) in stage I–III CRC patients.

## Methods

### Study population

For this study, we used data of the ongoing COLON study: *Co*lorectal cancer: *L*ongitudinal, *O*bservational study on *N*utritional and lifestyle factors that may influence colorectal tumor recurrence, survival, and quality of life [18]. In eleven participating hospitals, hospital staff invited eligible patients to participate in the COLON study shortly after diagnosis and before scheduled surgery. Patients were not eligible if they had a history of CRC, a previous (partial) bowel resection, known hereditary CRC, inflammatory bowel disease, dementia, or another mental condition limiting their ability to fill out surveys, or were non-Dutch speaking. For the present study, participants diagnosed between 2010 and 2015, from seven of the eleven participating hospitals, were included. For the participants from the other hospitals and participants included after 2015, no CT images had been retrieved from the medical records at time of the current analyses. Exclusion criteria for the present analyses were missing data on fatigue, height, stage of disease, or comorbidities; stage IV CRC; and missing or unusable CT images (i.e., CT images of poor quality or scans where muscle tissue was partly cut off). In addition, we only used data of patients who had a pretreatment CT image performed no longer than 3 months before diagnosis, as we considered that to be representative for body composition at diagnosis.

The COLON study was approved by the Committee on Research involving Human Subjects, region Arnhem–Nijmegen, the Netherlands, and all study participants provided written informed consent.

### Fatigue

Fatigue was assessed using the European Organization for Research and Treatment of Cancer Quality of Life Questionnaire C30 (EORTC QLQ-C30) version 3.0 [19] at three points in time: at diagnosis and 6 and 24 months after diagnosis. In the EORTC QLQ-C30, the fatigue subscale is comprised of three items (During the past week: Did you need to rest?, Have you felt weak?, Were you tired?), with four response options used to score the items: “Not at all,” “A little,” “Quite a bit,” and “Very much.” The raw score for fatigue was linearly transformed into a score of 0–100 points as described earlier [19], with a higher score indicating higher levels of fatigue. A score of > 39 was defined as having clinically relevant fatigue as recommended elsewhere [20, 21].

### Body composition

Cross-sectional areas (cm^2^) of skeletal muscle, VAT, and SAT were assessed using standard radiodensity thresholds measured in Hounsfield units (HU) in preoperative CT images at the level of the 3rd lumbar vertebrae using Slice-O-Matic 5.0 (Tomovision, Montreal, Canada). For skeletal muscle, the threshold values were between − 29 and + 150 HU, for VAT between − 150 and − 50 HU, and for SAT between − 190 and − 30 HU [22, 23]. The skeletal muscle cross-sectional area was adjusted for height squared (cm^2^/m^2^) to calculate skeletal muscle index (SMI). SMR was assessed as the mean radiodensity of the total skeletal muscle cross-sectional area at the level of the 3rd lumbar vertebrae.

### Demographic, lifestyle, and clinical data

Demographic information including age at diagnosis, sex, and weight and height was collected using self-administered questionnaires. Physical activity was assessed at diagnosis, 6, and 24 months post-diagnosis using the validated Short QUestionnaire to ASsess Health-enhancing physical activity (SQUASH) [24]. Data on stage of disease, tumor site, treatment, comorbidities, complications after surgery, and stoma placement after surgery were retrieved from the Dutch ColoRectal Audit [25].

### Power calculation and data analysis

We calculated that 600 patients were needed to have 80% power to detect a prevalence ratio of larger than 1.6 or smaller than 0.6, assuming that 40% of the population would suffer from fatigue [4, 26] and an alpha of 0.05.

Demographic, clinical, and lifestyle characteristics are presented as mean and standard deviation (SD) for continuous variables with normal distribution, median, and interquartile range (IQR) for continuous variables without normal distribution or frequency and percentage for categorical variables. Differences in patient characteristics were analyzed using the independent *T* test (continuous variables) or Pearson *χ*^2^ test (categorical variables).

Restricted cubic spline (RCS) analyses [27] were used to investigate associations of SMI, SMR, VAT, and SAT with fatigue at all timepoints and to assess whether associations were non-linear. Knots were placed at the 5th, 50th, and 95th percentiles of SMI, SMR, VAT, or SAT. Prevalence ratios (PR) and 95% confidence intervals (CI) for the associations of SMI, SMR, VAT, and SAT with fatigue were estimated using RCS functions in Cox proportional hazard regression models with a fixed timepoint. PRs were chosen instead of odd ratios since the latter tend to overestimate the size of the association when the outcome is common [28]. Median value for each body composition parameter was set as the reference in each model. As a complement to the graphic presentation of the RCS graphs, PRs with 95% confidence intervals for specific SMI, SMR, VAT, and SAT values were calculated using the RCS analyses.

All analyses were tested for effect modification by gender, by calculating the *p* value for interaction. In none of the analyses, gender was identified as effect modifier. Potential confounders were included in the final model if they change the PR for fatigue with 10% or more when the variable was individually added to a crude model including SMI/SMR/VAT/SAT, age, stage of disease, and gender. For all analyses, potential confounders were SMI (continuous, in the model for SMR, VAT, and SAT), SMR (continuous, in the model for SMI, VAT, and SAT), total adipose tissue (calculated as sum of VAT and SAT, continuous), comorbidities (0, 1, and ≥ 2), tumor site (colon/rectum), and physical activity (minutes per week of moderate-to-vigorous physical activity). For the analyses at 6 and 24 months, additional potential confounders were chemotherapy (yes/no), radiotherapy (yes/no), stoma placement after surgery (yes/no), and complications post-surgery (yes/no).

The statistical significance level for the analyses was set at *p* < 0.05. Statistical analyses were carried out using IBM SPSS v.23 (SPSS, Chicago, IL).

## Results

### Study population

**Fig. 1:**
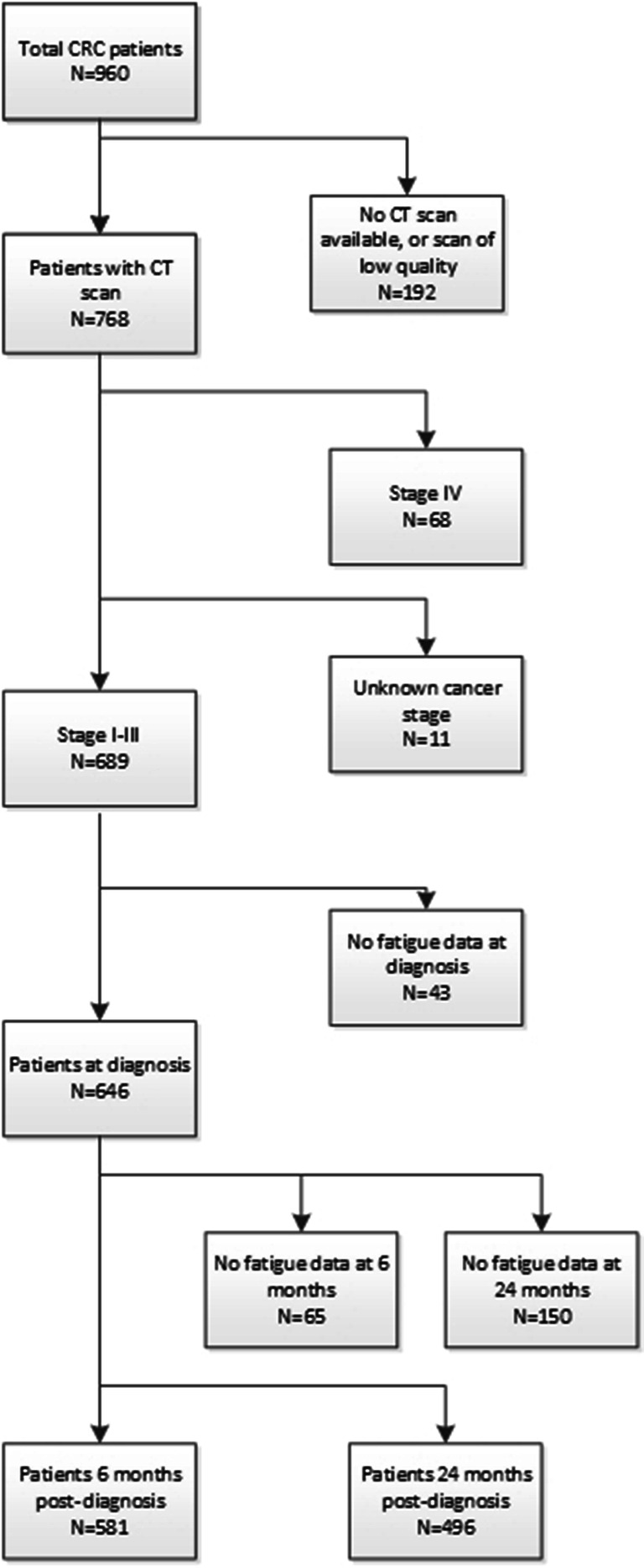
Flowchart of the inclusion process of patients in this study

The study population consisted of 960 patients, of which we had to exclude 192 patients because no suitable CT image was available; 68 patients because of stage IV disease; 11 patients because of missing information on stage of disease; and 63 patients because no fatigue data at diagnosis were available (Fig. 1). This resulted in a final dataset of 646 patients with fatigue data at diagnosis, 581 patients with also fatigue data at 6 months, and 496 patients with fatigue data at 24 months.

Baseline characteristics of the total study population and of patients with and without fatigue at the various timepoints are presented in Table 1. The average age of the total study population at diagnosis was 66.1 ± 8.8 years, 63% of the patients were men, and in 66% of the patients the tumor was located in the colon. The majority of the patients had stage III disease (46%), while the percentages of patients with stage I or II disease were 27% and 28%, respectively. At diagnosis, and at 6 and 24 months post-diagnosis, 18%, 25%, and 12% of the patients, respectively, experienced fatigue. Supplementary Tables 1 and 2 describe baseline characteristics by tertiles of SMI/SMR/VAT/SAT.

**Table 1:** Characteristics of stage I–III colorectal cancer patients with or without fatigue at diagnosis, 6 months post-diagnosis, and 24 months post-diagnosis

Fatigue										
	Total population at diagnosis	Diagnosis			6 months post-diagnosis			24 months post-diagnosis		
	*n* = 646	Yes *n* = 114 (18%)	No *n* = 532 (82%)	*p* value	Yes *n* = 148 (25%)	No *n* = 433 (75%)	*p* value	Yes *n* = 61 (12%)	No *n* = 435 (88%)	*p* value
Age at diagnosis (years, mean (SD))	66.1 (8.8)	66.3 (10.1)	66.0 (8.6)	0.74	63.4 (10.3)	66.6 (8.3)	< 0.01	66.0 (11.6)	65.5 (8.4)	0.72
Men (*n* (%))	406 (63)	61 (54)	345 (65)	0.02	86 (58)	283 (65)	0.09	42 (69)	270 (62)	0.03
Cancer stage (*n* (%))				0.13			< 0.01			0.18
I	174 (27)	26 (23)	148 (28)		17 (12)	144 (33)		15 (25)	133 (31)	
II	178 (28)	40 (35)	138 (26)		22 (15)	139 (32)		13 (21)	124 (29)	
III	294 (46)	48 (42)	246 (46)		109 (74)	150 (35)		3 (54)	178 (41)	
Tumor location (n (%))				< 0.01			0.27			0.61
Colon	426 (66)	94 (83)	332 (62)		92 (62)	288 (67)		37 (61)	282 (65)	
Rectal	220 (34)	20 (18)	200 (38)		56 (38)	145 (34)		24 (39)	153 (35)	
Number of comorbidities at diagnosis (*n* (%))				0.11			0.13			< 0.01
0	230 (36)	34 (30)	196 (37)		50 (34)	163 (38)		17 (28)	168 (39)	
1	167 (26)	26 (23)	141 (27)		30 (20)	116 (27)		9 (15)	113 (26)	
≥ 2	246 (38)	53 (47)	193 (36)		67 (45)	153 (35)		34 (56)	153 (35)	
Radiotherapy (*n* (%))^a^				< 0.01			0.01			0.19
Yes	171 (26)	16 (14)	152 (29)		50 (34)	104 (24)		21 (35)	114 (26)	
No	490 (74)	97 (86)	376 (71)		97 (66)	325 (75)		39 (65)	319 (74)	
Chemotherapy (*n* (%))^b^				0.21			< 0.01			0.52
Yes	191 (29)	38 (33)	145 (28)		74 (50)	87 (20)		18 (30)	113 (26)	
No	471 (71)	76 (67)	383 (73)		73 (50)	344 (79)		43 (70)	320 (74)	
Physical activity (moderate-vigorous min/week, median (IQR))^c^										
Diagnosis	600 (285–1110)	505 (229–983)	630 (300–1140)	0.21						
6 months					265 (80–540)	500 (240–870)	< 0.01			
24 months								412 (118–840)	570 (330–1020)	0.05
Skeletal muscle index (cm^2^/m^2^, mean (SD))	47.7 (8.7)	46.4 (7.8)	48.1 (8.8)	0.06	47.4 (8.9)	48.0 (8.6)	0.39	48.2 (7.6)	47.9 (8.7)	0.72
Skeletal muscle radiodensity (HU, mean (SD))	37.0 (8.2)	36.2 (8.4)	37.3 (8.2)	0.17	35.8 (9.3)	37.7 (7.8)	0.01	35.5 (8.2)	37.8 (7.9)	0.03
Visceral adipose tissue (cm^2^, median (IQR))	142 (73–217)	131 (78–204)	146 (74–219)	0.61	140 (72–215)	145 (76–215)	0.66	174 (96–238)	135 (65–208)	0.05
Subcutaneous adipose tissue (cm^2^, median (IQR))	163 (118–214)	162 (117–251)	163 (119–212)	0.61	183 (129–236)	157 (116–203)	< 0.01	177 (129–235)	163 (118–210)	0.35

^a^At diagnosis data of 5 patients missing; ^b^at diagnosis data of 4 patients missing; ^c^at diagnosis data of 7 patients missing. *SD*, standard deviation; *IQR*, interquartile range; *HU*, Hounsfield unit

### Difference between patients with and without fatigue

At diagnosis, age was similar for patients with and without fatigue, women more often reported fatigue, and patients with colon cancer more often experienced fatigue (Table 1). At this timepoint, patients with fatigue generally had more SAT.

Six months post-diagnosis, the patients who experienced fatigue were slightly younger, more often women, and received more often chemotherapy and/or radiotherapy. At 6 months post-diagnosis, the percentage of patients with colon cancer was almost similar between the group with and without fatigue. SMR was lower among patients with fatigue and patients with fatigue had more SAT.

At 24 months post-diagnosis, age was similar for patients with or without fatigue and the percentage of men was higher among patients with fatigue. The percentage of patients who received chemotherapy was almost similar between the group with and without fatigue, while slightly more patients with fatigue 24 months post-diagnosis received radiotherapy. At 24 months, SMR was lower among patients with fatigue and patients with fatigue had more VAT.

At all three timepoints, the number of hours of moderate-to-vigorous physical activity was lower among the patients with fatigue, and the percentage of patients with two or more comorbidities at diagnosis was higher among patients with fatigue.

### Association between body composition and fatigue

**Fig. 2:**
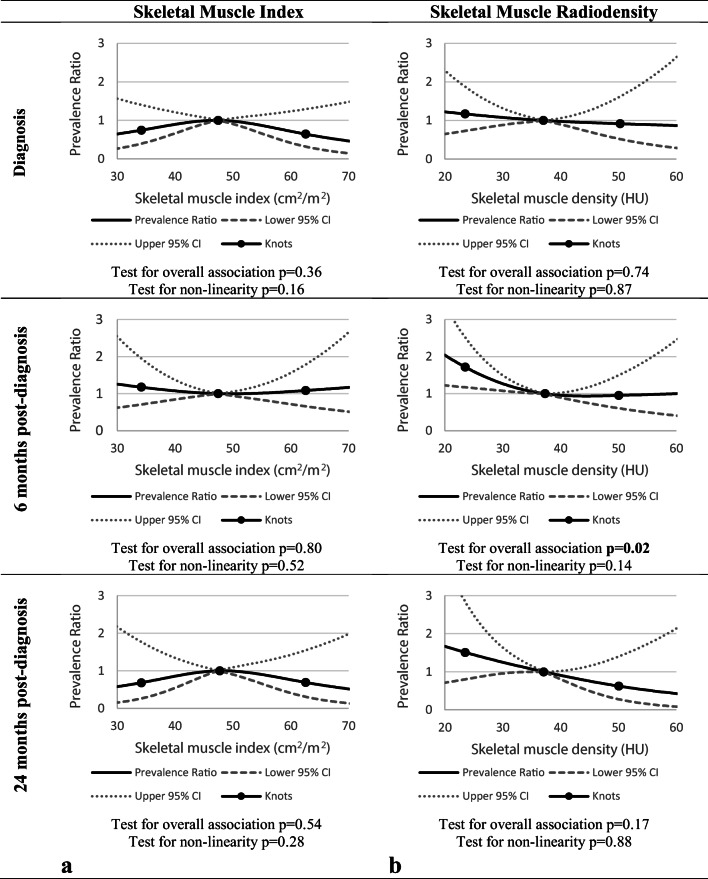
The association between skeletal muscle index (SMI) (**a**), skeletal muscle radiodensity (SMR) (**b**), and fatigue at diagnosis, at 6 months, and 24 months post-diagnosis, adjusted for age, gender, and stage of disease (SMI at all timepoints; SMR at diagnosis) and age, gender, stage of disease and physical activity (minutes per week of moderate-to-vigorous physical activity, for SMR at 6 and 24 months post-diagnosis) with three knots located at the 5th, 50th (reference), and 95th percentiles of the distribution of SMI or SMR

No association was observed between SMI and fatigue at any of the three timepoints (test for overall association at diagnosis and at 6 and 24 months post-diagnosis: *p* = 0.36, *p* = 0.80, and *p* = 0.54, respectively) (Fig. 2a). PRs with 95% confidence intervals for specific SMI values can be found in Table 2.

**Table 2:** Estimated prevalence ratios for specific skeletal muscle index, skeletal muscle radiodensity, and visceral adipose tissue and subcutaneous adipose tissue values and fatigue, with median values as the reference category (i.e., for skeletal muscle index: 47.5 cm^2^/m^2^; for skeletal muscle radiodensity: 37.2 HU; for visceral adipose tissue: 142.6 cm^2^; for subcutaneous adipose tissue: 162.9 cm^2^)

	Diagnosis		6 months		24 months	
	PR	95% CI	PR	95% CI	PR	95% CI
SMI (cm^2^/m^2^)^*^						
30	0.65	0.27–1.56	1.25	0.62–2.54	0.58	0.15–2.17
40	0.90	0.66–1.21	1.07	0.84–1.37	0.86	0.54–1.34
50	0.98	0.91–1.06	1.00	0.94–1.06	0.99	0.90–1.09
60	0.72	0.42–1.24	1.06	0.72–1.56	0.76	0.41–1.42
SMR (HU)^**^						
20	1.22	0.65–2.30	*2.04*	*1.22–3.40*	1.67	0.71–3.90
30	1.08	0.88–1.32	*1.27*	*1.08–1.49*	1.25	0.96–1.64
40	0.98	0.90–1.06	0.96	0.89–1.02	0.91	0.81–1.02
50	0.92	0.53–1.60	0.95	0.61–1.49	0.63	0.28–1.40
VAT (cm^2^)^***^						
50	0.90	0.61–1.32	1.14	0.82–1.60	0.60	0.34–1.07
150	1.00	0.98–1.02	1.00	0.98–1.01	1.03	1.00–1.06
250	0.92	0.71–1.19	1.04	0.86–1.25	1.13	0.82–1.55
350	0.75	0.40–1.43	1.20	0.77–187	0.95	0.44–2.02
SAT (cm^2^)^***^						
50	1.41	0.82–2.41	0.87	0.52–1.47	0.55	0.23–1.31
150	1.02	0.97–1.06	0.99	0.94–1.03	0.95	0.89–1.02
250	1.12	0.94–1.34	1.10	0.93–1.31	1.14	0.85–1.53
350	*1.70*	*1.20–2.42*	1.22	0.87–1.72	1.04	0.51–2.11

*Adjusted for age, gender, and stage of disease at all timepoints. **At diagnosis adjusted for age, gender, and stage of disease; at 6 and 24 months additionally adjusted for physical activity (minutes per week of moderate-to-vigorous physical activity). ***Adjusted for age, gender, and stage of disease and skeletal muscle radiodensity at all timepoints. *PR*, prevalence ratio; *95% CI*, 95% confidence interval; *SMI*, skeletal muscle index; *SMR*, skeletal muscle radiodensity; *VAT*, visceral adipose tissue; *SAT*, subcutaneous adipose tissue

A significant linear association between SMR and fatigue was observed at 6 months post-diagnosis (test for overall association *p* = 0.02, test for non-linearity *p* = 0.14), where lower levels of SMR were associated with higher prevalence of fatigue (see Fig. 2b). Table 2 shows that relative to the median, SMR levels below the median were significantly associated with higher fatigue prevalence. No significant associations were observed between SMR and fatigue at diagnosis (test for overall association *p* = 0.74) and at 24 months post-diagnosis (test for overall association at diagnosis *p* = 0.17).

**Fig. 3:**
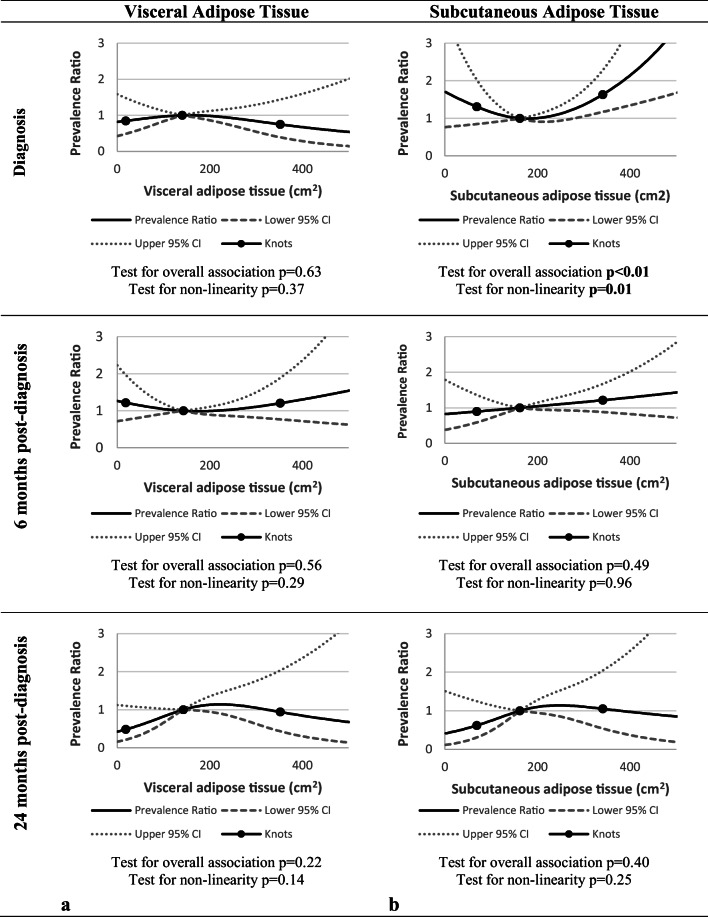
The association between visceral adipose tissue (VAT) (**a**), subcutaneous adipose tissue (SAT) (**b**), and fatigue at diagnosis, at 6 months and 24 months post-diagnosis, adjusted for age, gender, stage of disease, and skeletal muscle radiodensity at all timepoints, with three knots located at the 5th, 50th (reference), and 95th percentiles of the distribution of VAT or SAT

For VAT, no significant association with fatigue was observed at any of the timepoints (test for overall association at diagnosis and at 6 and 24 months post-diagnosis: *p* = 0.63, *p* = 0.56, and *p* = 0.22, respectively) (see Fig. 3a).

Levels of SAT were non-linearly associated with fatigue at diagnosis, where higher levels of SAT were significantly associated with higher prevalence of fatigue (test for overall association *p* < 0.01, test for non-linearity *p* = 0.01) (see Fig. 3b). Table 2 also illustrates that higher SAT levels relative to the median were associated with higher fatigue prevalence at baseline. At both 6 and 24 months, SAT was not associated with fatigue (test for overall association at 6 and 24 months post-diagnosis: *p* = 0.63 and *p* = 0.22, respectively).

## Discussion

The present study is the first study to investigate associations of body composition at time of diagnosis with fatigue at diagnosis, and 6 and 24 months post-diagnosis in stage I–III CRC patients. Having more SAT at diagnosis was associated with higher prevalence of fatigue at diagnosis, while lower SMR levels at diagnosis were associated with fatigue at 6 months post-diagnosis.

We did not observe an association between low SMI and fatigue at diagnosis, while such an association of low SMI and more fatigue at cancer diagnosis was observed among men in two earlier studies among different cancer populations [15, 16]. Those two studies included mainly late-stage cancer patients, while the present study included stage I–III CRC patients. In late-stage cancer patients, low SMI might be more often the result of tumor-induced muscle degradation, while low SMI among stage I–III is most likely age and lifestyle related since cachexia is less common within patient group among these earlier stages [29]. Since fatigue is more prevalent among late-stage cancer patients [5], the association between low SMI and fatigue among late-stage patients might be driven by the progressive tumor instead of low SMI itself. This might explain why no association was observed at diagnosis in the present study. In the present study, we also did not observe an association between SMI at diagnosis and fatigue 2 years after diagnosis. This is similar to what was observed in a study of van Roekel et al. [17]. In that study among stage I–III CRC patients, SMI was not associated with fatigue 2–10 years post-diagnosis.

In the present study, we observed that lower SMR levels were associated with higher prevalence of fatigue 6 months post-diagnosis, but not at diagnosis or 24 months post-diagnosis. Nevertheless, at 24 months, the shape of the RCS appeared similar to the shape of the association at 6 months, but did not reach statistical significance at 24 months. This may be the result of lower statistical power at the 24 month timepoint: at 24 months, the total number of participants in the dataset was lower than at the earlier timepoints, while the prevalence of fatigue at this timepoint was also lower than at the earlier timepoints. Upon diagnosis, fatigue may partly be cancer-related, but may also partly be pre-existing general fatigue, but we could not differentiate between the two in our study. At the 6 month timepoint, most patients will have completed the cancer treatment or will be about to complete chemotherapy. Therefore, at that point in time, fatigue is likely to be more cancer-related than upon diagnosis. As the underlying reasons for fatigue may differ between timepoints, the mechanisms and associations with body composition and SMR may also differ.

In our study, lower SMR was associated with more fatigue; prior research among cancer survivors have linked lower SMR to other outcomes, including worse physical function [30] and higher mortality [31–38]. This link may potentially partly be explained by increased pro-inflammatory cytokine production, as this has been reported with lower levels of SMR [39]. Those pro-inflammatory factors may increase the risk of fatigue. Another explanation might be that lower levels of SMR are usually seen in patients with an inferior overall condition (i.e., multiple comorbidities, higher ASA score) [40, 41]. Because of this, patients with low SMR may experience more (long-term) side effects from treatment. Further studies are needed to understand the mechanisms behind the association of low SMR with fatigue.

To the best of our knowledge, the present study was the first study to investigate the association of SAT with fatigue among cancer patients. The results from our study suggest that at time of diagnosis, high levels of SAT are associated with higher prevalence of fatigue. Upon diagnosis, fatigue may partly be cancer-related, but can also partly be pre-existing general fatigue. In the general population, more adiposity has been found to be associated with more physical fatigue, which has been attributed to higher adiposity giving rise to chronic low-grade inflammation. But, also, there data are not fully consistent [42]. Interestingly, the association was found only for SAT and not for VAT. This was somewhat unexpected, since VAT is known to be more metabolically active than SAT [43] and a higher inflammatory response seen with higher levels of VAT could have explained a potential association with fatigue. A potential reason for this is that the assessment of VAT from CT images may be less accurate than the assessment of SAT. Several authors have questioned the use of a single slice CT image to assess VAT because the natural movement of abdominal soft tissue might influence the amount of VAT shown at the level of L3 [44, 45]. This may result in higher variation and, therefore, less statistical power to detect associations.

A limitation of the study was that body composition data were only available at diagnosis; as in the Netherlands, CT images are only standard of care for diagnosis/staging, but not in the period of follow-up [46]. Therefore, we could not assess how SMI, SMR, VAT, and/or SAT change post-diagnosis and whether any changes impact the association with fatigue. A second limitation was that statistical power was lower at 24 months post-diagnosis than at the earlier timepoints. Strengths of this study were the use of CT images to assess body composition and the availability of fatigue data at multiple timepoints.

In conclusion, having more SAT was associated with more fatigue at diagnosis, while low levels of SMR were associated with more fatigue at 6 months post-diagnosis. Studies with longitudinal measurements of body composition and fatigue are needed to further increase our understanding on this topic and to investigate potential mechanisms. In addition, this suggests that it may be interesting to investigate whether interventions that aim to increase SMR around the time of diagnosis may help to lower fatigue.

## Electronic supplementary material

ESM 1 (DOCX 30 kb)
