# Supplementary material for: Body composition and its association with fatigue in the first 2 years after colorectal cancer diagnosis
Source: J Cancer Surviv. 2020 Oct 17;15(4):597–606. doi: 10.1007/s11764-020-00953-0 (PMC8272706; doi:10.1007/s11764-020-00953-0)
Supplement: Supplementary file 1 — (DOCX 30 kb) [file 11764_2020_953_MOESM1_ESM.docx]

Supplementary Table 1-2 present baseline characteristics of stage I-III colorectal cancer patients by body composition tertiles.

**Table 1:** Baseline characteristics by skeletal muscle index and skeletal muscle radiodensity tertiles

**Table 2:** Baseline characteristics by visceral adipose tissue and subcutaneous adipose tissue tertiles

**Supplementary Table 1:** Characteristics of stage I-III colorectal cancer patients by skeletal muscle index (SMI) and skeletal muscle radiodensity (SMR) tertiles

|  | Total population at diagnosis | SMI | | | | SMR | | | |
| --- | --- | --- | --- | --- | --- | --- | --- | --- | --- |
|  | n=646 | Tertile 1  n=222 | Tertile 2 n=222 | Tertile 3  n=222 | P-value | Tertile 1  n=223 | Tertile 2 n=222 | Tertile 3  n=221 | P-value |
| Age at diagnosis [yrs, mean (SD)] | 66.1 (8.8) | 69.9 (8.3) | 65.8 (8.6) | 63.9 (9.2) | <0.01 | 70.3 (7.4) | 65.8 (7.9) | 62.2 (9.4) | <0.01 |
| Men [n (%)] | 406 (63) | 138 (62) | 138 (62) | 138 (62) | 1.00 | 139 (62) | 138 (62) | 137 (62) | 1.00 |
| Cancer stage [n (%)] |  |  |  |  | 0.08 |  |  |  | 0.74 |
| I | 174 (27) | 49 (22) | 59 (27) | 70 (32) |  | 57 (26) | 55 (25) | 66 (30) |  |
| II | 178 (28) | 75 (34) | 58 (26) | 53 (24) |  | 66 (30) | 62 (28) | 58 (26) |  |
| III | 294 (46) | 98 (44) | 105 (47) | 99 (45) |  | 100 (45) | 105 (47) | 97 (44) |  |
| Tumor location [n (%)] |  |  |  |  | 0.41 |  |  |  | 0.07 |
| Colon | 426 (66) | 152 (69) | 151 (68) | 140 (63) |  | 153 (69) | 156 (70) | 134 (61) |  |
| Rectal | 220 (34) | 70 (32) | 71 (32) | 82 (37) |  | 70 (31) | 66 (30) | 87 (40) |  |
| Number of comorbidities at diagnosis [n (%)] |  |  |  |  | 0.15 |  |  |  | <0.01 |
| 0 | 230 (36) | 85 (39) | 87 (39) | 66 (30) |  | 55 (25) | 81 (37) | 102 (46) |  |
| 1 | 167 (26) | 53 (24) | 60 (27) | 60 (27) |  | 56 (25) | 58 (26) | 59 (27) |  |
| ≥ 2 | 246 (38) | 81 (37) | 34 (34) | 96 (43) |  | 110 (50) | 83 (37) | 59 (27) |  |
| Radiotherapy [n (%)]^a^ |  |  |  |  | 0.56 |  |  |  | 0.49 |
| Yes | 171 (26) | 52 (23) | 58 (26) | 61 (28) |  | 55 (25) | 53 (24) | 63 (29) |  |
| No | 490 (74) | 170 (77) | 162 (73) | 158 (71) |  | 168 (75) | 166 (75) | 156 (71) |  |
| Chemotherapy [n (%)]^b^ |  |  |  |  | 0.49 |  |  |  | 0.30 |
| Yes | 191 (29) | 60 (27) | 70 (32) | 61 (28) |  | 60 (27) | 72 (33) | 59 (27) |  |
| No | 471 (71) | 161 (73) | 150 (68) | 160 (72) |  | 162 (73) | 148 (67) | 161 (73) |  |
| Physical activity [moderate-vigorous min/wk, median (IQR)]^c^ |  |  |  |  |  |  |  |  |  |
| Diagnosis | 600  (285-1110) | 645 (305-1148) | 630 (240-1075) | 525 (295-1125) | 0.32 | 560 (210-990) | 673 (360-1192) | 613 (300-1140) | 0.43 |
| 6 months | 435 (195-820) | 450 (210-780) | 420 (195-805) | 435 (183-851) | 0.97 | 383 (150-720) | 488 (255-870) | 420 (210-840) | 0.03 |
| 24 months | 550 (300-1009) | 578 (300-985) | 570 (270-1065) | 510 (284-960) | 0.38 | 480 (250-870) | 600 (360-1145) | 595 (270-1080) | 0.07 |
| Skeletal muscle index [cm^2^/m^2^, mean (SD)] | 47.7 (8.7) | 40.5 (5.7) | 47.4 (5.6) | 55.2 (7.5) | <0.01 | 46.1 (8.8) | 47.7 (8.5) | 49.4 (8.5) | <0.01 |
| Skeletal muscle radiodensity [HU, mean (SD)] | 37.0 (8.2) | 35.2 (8.4) | 37.1 (8.1) | 38.9 (7.8) | <0.01 | 28.3 (5.2) | 37.0 (2.6) | 45.8 (4.0) | <0.01 |
| Visceral adipose tissue [cm^2^, median (IQR)] | 142 (73-217) | 107 (62-186) | 138 (67-201) | 172 (92-265) | <0.01 | 182 (103-266) | 147 (85-208) | 94 (45-173) | <0.01 |
| Subcutaneous adipose tissue [cm^2^, median (IQR)] | 163 (118-214) | 148 (105-190) | 161 (117-213) | 182 (136-241) | <0.01 | 179 (137-240) | 163 (123-213) | 140 (102-190) | <0.01 |

^a^ = at diagnosis data of 5 patients missing; ^b^ = at diagnosis data of 4 patients missing; ^c^ = at diagnosis data of 7 patients missing

**Supplementary Table 2:** Characteristics of stage I-III colorectal cancer patients by visceral adipose tissue (VAT) and subcutaneous adipose tissue (SAT) tertiles

|  | Total population at diagnosis | VAT | | | | SAT | | | |
| --- | --- | --- | --- | --- | --- | --- | --- | --- | --- |
|  | n=646 | Tertile 1  n=222 | Tertile 2 n=222 | Tertile 3  n=222 | P-value | Tertile 1  n=220 | Tertile 2 n=220 | Tertile 3  n=226 | P-value |
| Age at diagnosis [yrs, mean (SD)] | 66.1 (8.8) | 64.8 (9.8) | 65.8 (9.3) | 67.8 (7.3) | <0.01 | 66.8 (8.5) | 67.0 (8.2) | 64.7 (9.8) | <0.01 |
| Men [n (%)] | 406 (63) | 138 (62) | 138 (62) | 138 (62) | 1.00 | 168 (77) | 142 (65) | 104 (46) | 1.00 |
| Cancer stage [n (%)] |  |  |  |  | 0.07 |  |  |  | 0.13 |
| I | 174 (27) | 47 (21) | 69 (31) | 62 (28) |  | 59 (27) | 60 (27) | 59 (26) |  |
| II | 178 (28) | 75 (34) | 53 (24) | 58 (26) |  | 69 (31) | 61 (28) | 56 (25) |  |
| III | 294 (46) | 100 (45) | 100 (45) | 102 (46) |  | 92 (42) | 99 (45) | 111 (49) |  |
| Tumor location [n (%)] |  |  |  |  | 0.07 |  |  |  | 0.66 |
| Colon | 443 (67) | 141 (64) | 141 (64) | 161 (73) |  | 135 (61) | 155 (71) | 153 (68) |  |
| Rectal | 223 (34) | 81 (37) | 81 (37) | 61 (28) |  | 85 (39) | 65 (30) | 73 (32) |  |
| Number of comorbidities at diagnosis [n (%)] |  |  |  |  | <0.01 |  |  |  | 0.01 |
| 0 | 230 (36) | 109 (49) | 83 (38) | 46 (21) |  | 91 (42) | 85 (39) | 62 (27) |  |
| 1 | 167 (26) | 52 (24) | 56 (26) | 65 (29) |  | 55 (25) | 52 (24) | 66 (29) |  |
| ≥ 2 | 246 (38) | 60 (27) | 81 (37) | 111 (50) |  | 72 (33) | 82 (37) | 98 (43) |  |
| Radiotherapy [n (%)]^a^ |  |  |  |  | 0.30 |  |  |  | 0.28 |
| Yes | 171 (26) | 64 (29) | 163 (74) | 50 (23) |  | 67 (31) | 47 (22) | 57 (25) |  |
| No | 490 (74) | 156 (71) | 163 (74) | 171 (77) |  | 152 (69) | 170 (78) | 168 (74) |  |
| Chemotherapy [n (%)]^b^ |  |  |  |  | 0.91 |  |  |  | 0.83 |
| Yes | 191 (29) | 66 (30) | 62 (28) | 63 (29) |  | 59 (27) | 65 (30) | 67 (30) |  |
| No | 471 (71) | 155 (70) | 159 (72) | 157 (71) |  | 159 (73) | 155 (71) | 157 (70) |  |
| Physical activity [moderate-vigorous min/wk, median (IQR)]^c^ |  |  |  |  |  |  |  |  |  |
| Diagnosis | 600  (818) | 790 (375-1220) | 562 (282-1080) | 502 (210-1010) | <0.01 | 667 (341-1228) | 600 (290-1095) | 522 (210-1065) | 0.36 |
| 6 months | 435 (625) | 450 (240-870) | 472 (210-803) | 420 (150-818) | 0.89 | 480 (230-900) | 450 (240-780) | 390 (136-795) | 0.20 |
| 24 months | 669 (709) | 660 (360-1140) | 510 (270-1013) | 480 (270-848) | 0.02 | 660 (308-1185) | 545 (304-985) | 450 (233-835) | 0.03 |
| Skeletal muscle index [cm^2^/m^2^, mean (SD)] | 47.7 (8.7) | 45.8 (7.8) | 47.6 (8.4) | 49.8 (9.4) | <0.01 | 47.4 (7.8) | 47.9 (8.9) | 47.8 (9.4) | <0.01 |
| Skeletal muscle radiodensity [HU, mean (SD)] | 37.0 (8.2) | 40.1 (7.9) | 37.5 (7.4) | 33.5 (8.1) | <0.01 | 40.3 (7.4) | 36.6 (7.5) | 34.3 (8.7) | <0.01 |
| Visceral adipose tissue [cm^2^, median (IQR)] | 142 (144) | 52 (30-92) | 156 (96-188) | 263 (184-313) | <0.01 | 79 (35-155) | 152 (88-219) | 181 (121-277) | <0.01 |
| Subcutaneous adipose tissue [cm^2^, median (IQR)] | 163 (97) | 121 (88-166) | 164 (128-210) | 197 (151-260) | <0.01 | 101 (83-118) | 163 (139-178) | 240 (206-305) | <0.01 |

^a^ = at diagnosis data of 5 patients missing; ^b^ = at diagnosis data of 4 patients missing; ^c^ = at diagnosis data of 7 patients missing
